# Antigen processing sites in gp120 are conserved across HIV virus clades

**DOI:** 10.1186/1742-4690-9-S2-P297

**Published:** 2012-09-13

**Authors:** B Yu, SM O’Rourke, JF Morales, GP Tatsuno, KA Mesa, PW Berman

**Affiliations:** 1 University of California, Santa Cruz, Santa Cruz, CA, USA

## Background

A puzzling observation in HIV vaccine research is the fact that recombinant gp120 is able to adsorb bNAbs from HIV+ patient sera, but unable to elicit such bNAbs. To account for its poor immunogenicity, we wondered if the epitopes recognized by bNAbs might be proteolyzed in vivo. Cathepsins L, S, and D are the major proteases responsible for antigen processing and presentation. Previously, we defined the cathepsin cleavage sites on MN rgp120 and found that they co-localized with epitopes recognized by bNAbs. Although examination of gp120 sequences suggested that these sites were conserved, the recognition motifs for cathepsins are poorly defined and it was important to verify their presence by protease digestion studies.

## Methods

Purified gp120s from three clades of HIV were digested with cathepsins L, S, and D. These included envelope proteins from the from the 108060 (clade B), A244 (CRF01_AE) and 97001 (clade C) isolates. N-terminal sequencing was used to identify the cathepsin cleavage sites.

## Results

When combined with the previous MN-rgp120 results, we found that 6 out of 10 cathepsin cleavage sites were conserved in four viruses from three different clades of HIV. We found that polymorphisms that inactivate cleavage sites often result in the formation of an alternate nearby site. Although cleavage is an ordered processing beginning in the V3 domain, the cleavage sites in this domain are more polymorphic than other sites.

## Conclusion

Our results suggest that cathepsin cleavage sites are highly conserved in gp120 and co-localize to regions recognized by bNAbs. These results are consistent with our hypothesis that the poor immunogenicity of gp120 epitopes results from protease digestion in vivo. Current studies are in progress to determine whether inactivation of these sites or enzymes may provide a new approach to improving the immunogenicity of epitopes recognized by bNAbs.

